# Supplementary material for: Hot spot prediction in protein-protein interactions by an ensemble system
Source: BMC Syst Biol. 2018 Dec 31;12(Suppl 9):132. doi: 10.1186/s12918-018-0665-8 (PMC6311905; doi:10.1186/s12918-018-0665-8)
Supplement: Supplementary file 2 — All relevant MATLAB code. A simple Matlab implement of our predictor. (ZIP 19,742 kb) [file 12918_2018_665_MOESM2_ESM.zip › Additional file 2/Read ASA/ASA website/ASEdb/1VFBB.htm]

 NetSurfP - Protein Surface Accessibility and Secondary Structure Predictions

  

|  |  |  |
| --- | --- | --- |
|  |  | NetSurfP - Protein Surface Accessibility and Secondary Structure Predictions  Technical University of Denmark |

  


---

```
# For publication of results, please cite:
# A generic method for assignment of reliability scores applied to solvent accessibility predictions.
# Bent Petersen, Thomas Nordahl Petersen, Pernille Andersen, Morten Nielsen and Claus Lundegaard
# BMC Structural Biology 2009, 9:51 doi:10.1186/1472-6807-9-51
#
# Column 1: Class assignment - B for buried or E for Exposed - Threshold: 25% exposure, but not based on RSA
# Column 2: Amino acid
# Column 3: Sequence name
# Column 4: Amino acid number
# Column 5: Relative Surface Accessibility - RSA
# Column 6: Absolute Surface Accessibility
# Column 7: Z-fit score for RSA prediction
# Column 8: Probability for Alpha-Helix
# Column 9: Probability for Beta-strand
# Column 10: Probability for Coil
E Q  1VFBB                  1    0.740 132.146   1.161   0.005   0.015   0.979
B V  1VFBB                  2    0.216  33.153  -0.262   0.004   0.616   0.381
E Q  1VFBB                  3    0.407  72.744   1.170   0.002   0.816   0.182
B L  1VFBB                  4    0.150  27.502   0.751   0.018   0.846   0.136
E Q  1VFBB                  5    0.381  67.957   0.569   0.021   0.756   0.223
B E  1VFBB                  6    0.240  41.858   0.721   0.022   0.552   0.426
E S  1VFBB                  7    0.382  44.724  -0.479   0.020   0.205   0.775
E G  1VFBB                  8    0.433  34.077  -1.700   0.018   0.088   0.893
E P  1VFBB                  9    0.522  74.072  -2.286   0.018   0.047   0.935
E G  1VFBB                 10    0.520  40.885  -1.785   0.019   0.141   0.840
E L  1VFBB                 11    0.355  64.909  -0.718   0.021   0.279   0.699
B V  1VFBB                 12    0.282  43.390   0.504   0.022   0.359   0.619
E A  1VFBB                 13    0.411  45.325   0.977   0.020   0.205   0.775
E P  1VFBB                 14    0.463  65.756  -0.668   0.018   0.047   0.935
E S  1VFBB                 15    0.603  70.660  -1.968   0.005   0.015   0.979
E Q  1VFBB                 16    0.449  80.174  -0.616   0.004   0.085   0.910
E S  1VFBB                 17    0.402  47.114  -0.599   0.004   0.420   0.576
B L  1VFBB                 18    0.145  26.568   0.731   0.002   0.816   0.182
E S  1VFBB                 19    0.397  46.540   0.842   0.001   0.900   0.099
B I  1VFBB                 20    0.070  12.987   0.541   0.001   0.900   0.099
B T  1VFBB                 21    0.132  18.253   0.878   0.001   0.900   0.099
B C  1VFBB                 22    0.042   5.939   0.248   0.001   0.900   0.099
B T  1VFBB                 23    0.252  34.994   0.467   0.001   0.900   0.099
B V  1VFBB                 24    0.083  12.772  -0.331   0.003   0.718   0.279
B S  1VFBB                 25    0.171  19.994  -0.627   0.004   0.420   0.576
B G  1VFBB                 26    0.246  19.360  -1.720   0.004   0.138   0.858
B F  1VFBB                 27    0.267  53.627  -1.022   0.004   0.197   0.799
B S  1VFBB                 28    0.176  20.662  -0.991   0.005   0.336   0.660
B L  1VFBB                 29    0.122  22.283  -0.704   0.022   0.359   0.619
B T  1VFBB                 30    0.208  28.794  -0.993   0.020   0.205   0.775
B G  1VFBB                 31    0.285  22.437  -1.061   0.018   0.088   0.893
B Y  1VFBB                 32    0.104  22.268   0.045   0.019   0.141   0.840
B G  1VFBB                 33    0.069   5.454   0.075   0.021   0.451   0.528
B V  1VFBB                 34    0.025   3.842   0.575   0.021   0.756   0.223
B N  1VFBB                 35    0.044   6.486  -0.144   0.018   0.846   0.136
B W  1VFBB                 36    0.031   7.552   0.620   0.018   0.846   0.136
B V  1VFBB                 37    0.035   5.410   0.216   0.018   0.846   0.136
B R  1VFBB                 38    0.105  24.091   0.683   0.021   0.756   0.223
B Q  1VFBB                 39    0.084  15.020   0.204   0.021   0.451   0.528
B P  1VFBB                 40    0.204  28.891   0.904   0.005   0.045   0.951
E P  1VFBB                 41    0.348  49.438   0.685   0.005   0.015   0.979
E G  1VFBB                 42    0.535  42.065  -1.556   0.018   0.019   0.964
E K  1VFBB                 43    0.410  84.255   0.497   0.018   0.019   0.964
E G  1VFBB                 44    0.452  35.580  -0.278   0.018   0.088   0.893
B L  1VFBB                 45    0.159  29.058   0.884   0.005   0.336   0.660
B E  1VFBB                 46    0.104  18.099  -0.266   0.003   0.718   0.279
B W  1VFBB                 47    0.046  11.111  -0.168   0.001   0.900   0.099
B L  1VFBB                 48    0.025   4.614   0.876   0.001   0.959   0.040
B G  1VFBB                 49    0.018   1.393  -0.139   0.001   0.959   0.040
B M  1VFBB                 50    0.113  22.691  -0.488   0.001   0.959   0.040
B I  1VFBB                 51    0.028   5.180   0.504   0.001   0.900   0.099
B W  1VFBB                 52    0.202  48.581   0.508   0.004   0.514   0.481
E G  1VFBB                 53    0.390  30.662  -1.099   0.004   0.138   0.858
E D  1VFBB                 54    0.624  89.962  -2.223   0.005   0.015   0.979
E G  1VFBB                 55    0.434  34.172  -2.053   0.018   0.019   0.964
E N  1VFBB                 56    0.467  68.413  -0.725   0.018   0.047   0.935
B T  1VFBB                 57    0.294  40.792  -0.149   0.019   0.141   0.840
E D  1VFBB                 58    0.402  57.971   0.290   0.020   0.205   0.775
B Y  1VFBB                 59    0.209  44.621   0.493   0.056   0.142   0.802
B N  1VFBB                 60    0.211  30.949   0.041   0.053   0.043   0.903
E S  1VFBB                 61    0.639  74.926  -0.451   0.053   0.043   0.903
E A  1VFBB                 62    0.409  45.094   0.117   0.052   0.084   0.864
B L  1VFBB                 63    0.085  15.527  -0.164   0.019   0.141   0.840
E K  1VFBB                 64    0.388  79.894   0.867   0.020   0.205   0.775
B S  1VFBB                 65    0.244  28.597  -1.521   0.005   0.262   0.733
B R  1VFBB                 66    0.211  48.319   0.723   0.003   0.718   0.279
B L  1VFBB                 67    0.052   9.521  -0.403   0.001   0.900   0.099
B S  1VFBB                 68    0.123  14.427   0.737   0.001   0.959   0.040
B I  1VFBB                 69    0.044   8.159   0.978   0.001   0.900   0.099
B S  1VFBB                 70    0.106  12.435   0.486   0.002   0.816   0.182
E K  1VFBB                 71    0.267  54.963   0.766   0.022   0.552   0.426
B D  1VFBB                 72    0.267  38.460  -0.266   0.005   0.336   0.660
E N  1VFBB                 73    0.453  66.305  -0.114   0.018   0.088   0.893
B S  1VFBB                 74    0.259  30.355  -1.558   0.018   0.047   0.935
E K  1VFBB                 75    0.526 108.219   0.910   0.056   0.142   0.802
E S  1VFBB                 76    0.263  30.859  -0.261   0.021   0.279   0.699
B Q  1VFBB                 77    0.195  34.881   0.856   0.021   0.756   0.223
B V  1VFBB                 78    0.073  11.159  -0.823   0.011   0.918   0.071
B F  1VFBB                 79    0.175  35.022   0.276   0.011   0.918   0.071
B L  1VFBB                 80    0.050   9.173   0.772   0.011   0.918   0.071
B K  1VFBB                 81    0.159  32.603  -0.325   0.018   0.846   0.136
B M  1VFBB                 82    0.082  16.408  -0.574   0.023   0.655   0.322
E N  1VFBB                 83    0.385  56.408  -0.174   0.021   0.279   0.699
E S  1VFBB                 84    0.402  47.091  -0.662   0.019   0.141   0.840
B L  1VFBB                 85    0.124  22.741   0.084   0.018   0.088   0.893
E H  1VFBB                 86    0.483  87.930   1.096   0.018   0.047   0.935
E T  1VFBB                 87    0.382  53.011  -0.457   0.052   0.084   0.864
E D  1VFBB                 88    0.467  67.352  -0.488   0.053   0.043   0.903
B D  1VFBB                 89    0.173  24.987  -0.509   0.052   0.084   0.864
B T  1VFBB                 90    0.110  15.299   0.338   0.022   0.359   0.619
B A  1VFBB                 91    0.037   4.044  -0.221   0.018   0.846   0.136
B R  1VFBB                 92    0.071  16.190   1.003   0.011   0.918   0.071
B Y  1VFBB                 93    0.036   7.693   0.895   0.011   0.918   0.071
B Y  1VFBB                 94    0.052  11.027   0.716   0.018   0.846   0.136
B C  1VFBB                 95    0.017   2.373   1.437   0.021   0.756   0.223
B A  1VFBB                 96    0.032   3.493  -0.233   0.023   0.655   0.322
B R  1VFBB                 97    0.142  32.632   0.261   0.021   0.279   0.699
E E  1VFBB                 98    0.316  55.240  -0.127   0.018   0.088   0.893
E R  1VFBB                 99    0.392  89.745  -0.931   0.018   0.047   0.935
E D  1VFBB                100    0.512  73.765  -2.385   0.005   0.015   0.979
B Y  1VFBB                101    0.302  64.516  -1.429   0.018   0.019   0.964
B R  1VFBB                102    0.176  40.281  -1.242   0.018   0.088   0.893
B L  1VFBB                103    0.092  16.900   0.034   0.018   0.088   0.893
B D  1VFBB                104    0.116  16.759  -0.359   0.019   0.141   0.840
B Y  1VFBB                105    0.088  18.870  -0.127   0.020   0.205   0.775
B W  1VFBB                106    0.048  11.568  -0.513   0.019   0.141   0.840
B G  1VFBB                107    0.049   3.825  -1.620   0.019   0.141   0.840
B Q  1VFBB                108    0.162  28.969   0.123   0.004   0.085   0.910
B G  1VFBB                109    0.099   7.760  -1.412   0.005   0.262   0.733
B T  1VFBB                110    0.086  11.998  -0.081   0.004   0.514   0.481
E T  1VFBB                111    0.262  36.339   0.110   0.001   0.900   0.099
B L  1VFBB                112    0.046   8.441   0.488   0.001   0.900   0.099
B T  1VFBB                113    0.140  19.418   0.120   0.001   0.900   0.099
B V  1VFBB                114    0.112  17.153  -0.443   0.002   0.816   0.182
E S  1VFBB                115    0.289  33.918  -0.857   0.004   0.514   0.481
E S  1VFBB                116    0.627  73.520  -1.885   0.003   0.003   0.994

Explain the output.  Go back.
```

---
